# Hypoxia-induced microRNA-10b-3p promotes esophageal squamous cell carcinoma growth and metastasis by targeting TSGA10

**DOI:** 10.18632/aging.102462

**Published:** 2019-11-26

**Authors:** Qiang Zhang, Jingjing Zhang, Zhanzhao Fu, Lixin Dong, Yong Tang, Chunlei Xu, Haifeng Wang, Tao Zhang, Yue Wu, Chao Dong, Shasha Shao, Guangxia Wang

**Affiliations:** 1 Department of Oncology, First Hospital of Qinhuangdao, Qinhuangdao, China; 2 Department of CT Scan Room, First Hospital of Qinhuangdao, Qinhuangdao, China; 3 Department of Gastroenterology, Affiliated Tumor Hospital of Xinjiang Medical University, Urumqi, China; 4 Department of Thoracic and Abdominal Radiotherapy, Affiliated Tumor Hospital of Xinjiang Medical University, Urumqi, China; 5 Department of Oncology, First Hospital of Lanzhou University, Lanzhou, China; 6 Department of Surgery, Traditional Chinese Hospital of Lu'an, Lu'an, Anhui, China; 7 Department of Oncology, First Hospital of Shijiazhuang, Shijiazhuang, China

**Keywords:** microRNAs, hypoxia, esophageal squamous cell carcinoma, metastasis

## Abstract

Evidence has shown that hypoxia promotes esophageal squamous cell carcinoma (ESCC) growth and metastasis, but the molecular mechanisms underlying that response remain poorly understood. MicroRNAs (miRNAs) are post-transcriptional regulators that participate in various cancer-related processes. Here, we demonstrated that hypoxia along with hypoxia-inducible factor 1α significantly increased expression of miR-10b-3p. Inhibition of miR-10b-3p weakened the effects of hypoxia on ESCC cell proliferation, migration and invasion, while miR-10b-3p overexpression had the opposite effects. Mechanistically, miR-10b-3p acted as cancer-promoting gene by targeting testis specific 10. Using a xenograft model, we observed that administration of miR-10b-3p agomir to tumors enhanced their growth and metastasis in vivo. These findings verified the potent regulatory role played by hypoxia-induced miR-10b-3p expression in ESCC progression. These results suggest that miR-10b-3p may be a useful therapeutic target for treating ESCC.

## INTRODUCTION

Esophageal cancer has a poor prognosis and is a major cause of cancer-related mortality due to its high metastatic potential [1]. Although esophageal adenocarcinoma now predominates in the western world, the majority of esophageal cancers in Asian countries are diagnosed as esophageal squamous cell carcinoma (ESCC) [2]. The overall survival rate among ESCC patients remains poor, despite advances in diagnostic technologies and therapies [3]. It is therefore crucial to understand the mechanisms underlying ESCC pathogenesis and progression and identify novel biomarkers and targets for ESCC diagnosis and treatment.

The modulatory roles played by microRNAs (miRNAs) in several cancer types have been previously reported [4, 5]. By suppressing the expression of their target genes, miRNAs can promote or inhibit both carcinogenesis and cancer progression [6]. In the present study, we focused on the effects of miR-10b-3p, which was previously shown to correlate with metastasis in breast cancer [6–9]. In addition, microRNA-10b-3p affects prognosis and the response to neo-adjuvant therapy in pancreatic ductal adenocarcinoma [10] and is predictive of survival in hepatocellular carcinoma patients treated with sorafenib [11]. In the present study, we examined the actions of miR-10b-3p in ESCC cells. Our findings suggest that hypoxia and hypoxia-inducible factor 1α (HIF-1α) enhance miR-10b-3p expression, which mediates ESCC cell growth by targeting testis specific 10 (TSGA10).

## RESULTS

### Hypoxia induces miR-10b-3p expression through HIF-1α in ESCC cells

**Figure 1:**
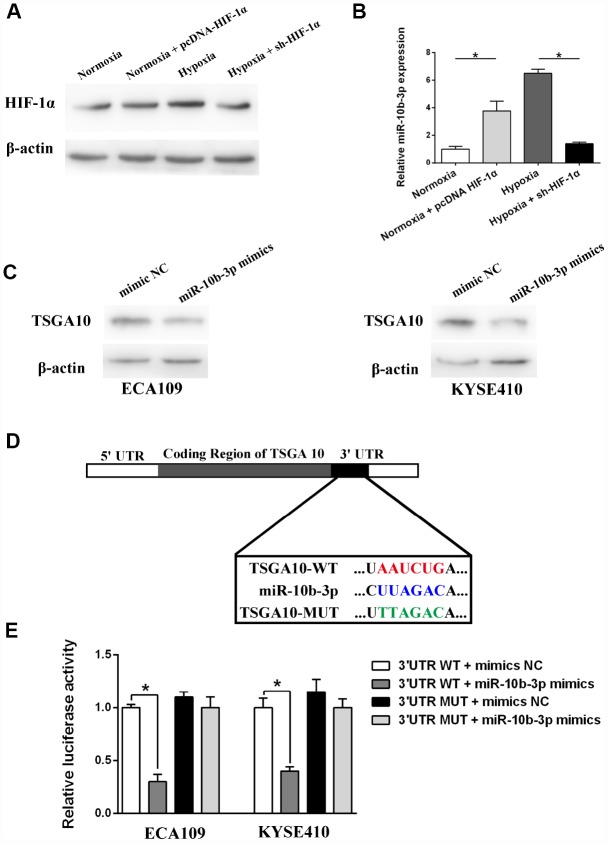
**Hypoxia induced miR-10b-3p expression and its target validation. ECA109 cells transfected with sh-HIF-1α or pcDNA- HIF-1α were incubated for 48 hours under normoxic or hypoxic conditions.** (**A**) Western blotting showed HIF-1α expression. (**B**) Quantification of miR-10b-3p expression using qRT-PCR. (**C**) ECA109 and KYSE410 cells were transfected with miR-10b-3p mimics or mimics NC, after which TSGA10 expression was assessed by western blotting. (**D**) Predicted miR-10b-3p wild-type binding sites (WT) or mutant binding sites (MUT) were cloned into a luciferase reporter. Cells co-transfected with miR-10b-3p mimics or controls and WT or MUT luciferase constructs were subjected to luciferase assay. (**E**) Measurement of luciferase activity. *P < 0.05.

ECA109 cells, a human ESCC line, were transfected with sh-HIF-1α or pcDNA-HIF-1α and then incubated for 48 hours under hypoxic and normoxic conditions, after which HIF-1α expression was assessed. As presented in Figure 1A, hypoxia enhanced HIF-1α expression, and this effect was blocked by sh-HIF-1α. On the other hand, pcDNA-HIF-1α transfection induced HIF-1α expression, even under normoxic conditions, which in turn led to increased miR-10b-3p expression (Figure 1B). Suppression of HIF-1α expression weakened the effect of hypoxia on miR-10b-3p. These data suggest that hypoxia increases miR-10b-3p expression through HIF-1α in ESCC cells.

### TSGA10 is a direct target of miR-10b-3p in ESCC cells

The algorithmic program-TargtScan was used to predict the targets of miR-10b-3p [12]. Based on the “Cumulative weighted context++ score,” TSGA10 was selected as the candidate target for further study. We initially showed that increasing miR-10b-3p suppressed TSGA10 expression in both ECA109 and KYSE410 (another ESCC line) cells, which suggests that TSGA10 may be a direct target of miR-10b-3p (Figure 1C). Dual-luciferase reporter assays were then performed to explore the interaction between miR-10b-3p and TSGA10. Fragments containing the wild-type (WT) miR-10b-3p binding sequence or a mutated sequence (MUT) in the 3′UTR region of TSGA10 mRNA were cloned into the downstream of a luciferase reporter (Figure 1D). Thereafter, the reporter along with miR-10b-3p mimics or mimics-NC (control) were co-transfected into ESCC cells. As shown in Figure 1E, miR-10b-3p overexpression decreased the luciferase activity, while mimics-NC had no significant effect on luciferase intensity. These results further confirm that TSGA10 is a direct target of miR-10b-3p in ESCC cells.

### MiR-10b-3p modulates ESCC proliferation under hypoxia by targeting TSGA10

**Figure 2:**
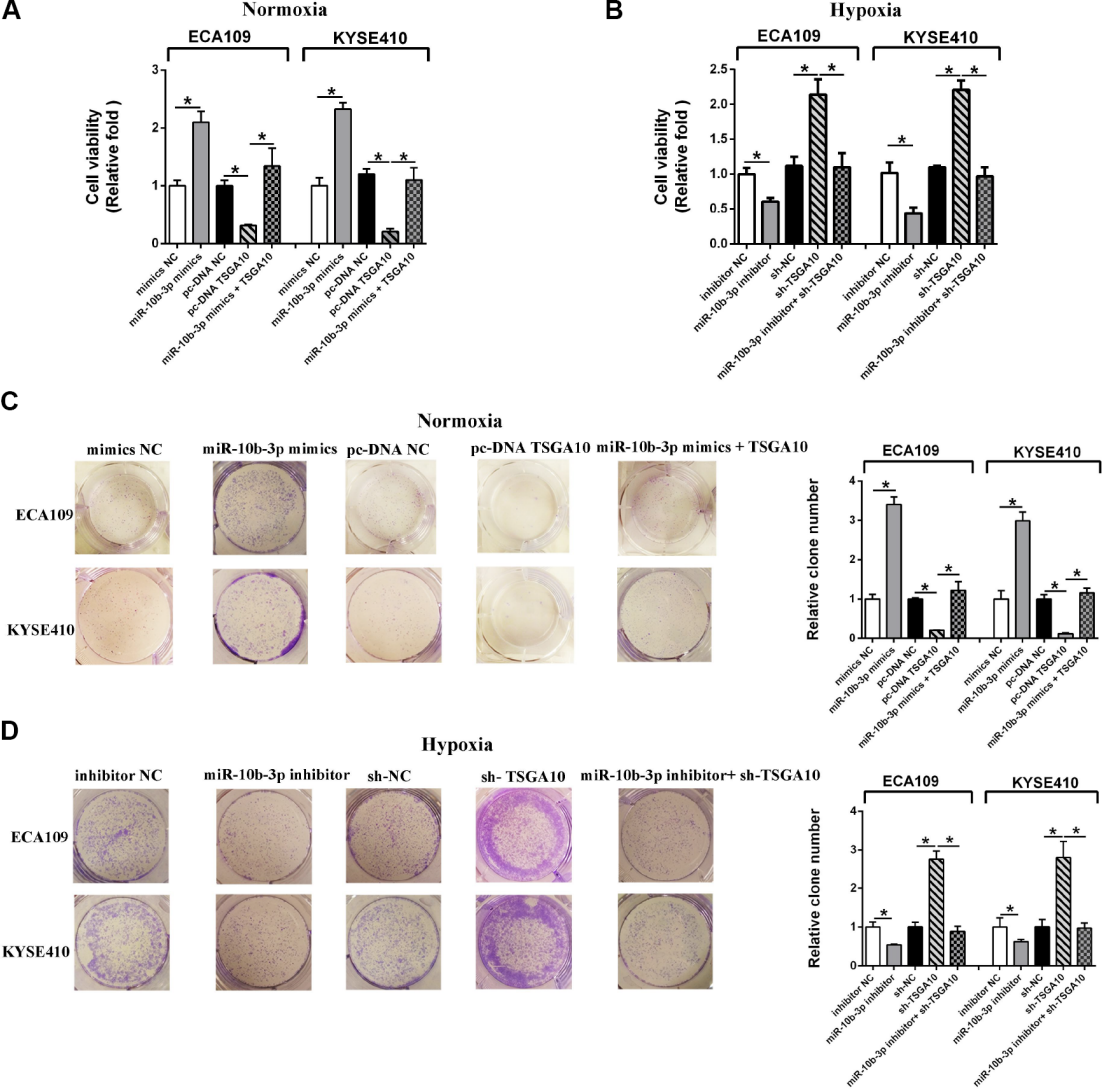
**Hypoxia-induced miR-10b-3p enhances ESCC cell proliferation.** (**A**) ECA109 and KYSE410 cells transfected with miR-10b-3p mimics or pc-DNA TSGA10 were cultured for 48 hours under normoxic conditions, after which cell viability was assessed. (**B**) ECA109 and KYSE410 cells transfected with miR-10b-3p inhibitor or sh-TSGA10 were cultured under hypoxic conditions, after which cell viability was assessed. (**C**) ECA109 and KYSE410 cells transfected with miR-10b-3p mimics or pc-DNA TSGA10 were cultured for 10 days under normoxic conditions, after which colonies were fixed, photographed and counted. (**D**) ECA109 and KYSE410 cells transfected with miR-10b-3p inhibitor or sh-TSGA10 were cultured for 10 days under hypoxic conditions, after which colonies were fixed, photographed, and counted. *P < 0.05.

CCK8 assays revealed that under normoxic conditions, miR-10b-3p overexpression promotes ESCC cell viability (Figure 2A). Similarly, miR-10b-3p overexpression also promoted colony formation by the cells (Figure 2C). A subsequent rescue experiment confirmed that TSGA10 is the functional target of miR-10b-3p in ESCC cells. As shown in Figure 2A and 2C, the enhanced ESCC cell proliferation stimulated by miR-10b-3p was reversed by up-regulation of TSGA10.

Hypoxia reportedly promotes cancer progression [13]. We therefore examined the participation of hypoxia induced miR-10b-3p in that effect. We observed that hypoxia promoted ESCC cell viability and proliferation, but those effects were reversed by suppressing miR-10b-3p (Figure 2B and 2D). Moreover, rescue experiments showed that under hypoxia TSGA10 knockdown restored the cell viability and proliferation that were suppressed by miR-10b-3p inhibition. (Figure 2D)

### Down-regulation of miR-10b-3p inhibits ESCC cell migration and invasion under hypoxia

**Figure 3:**
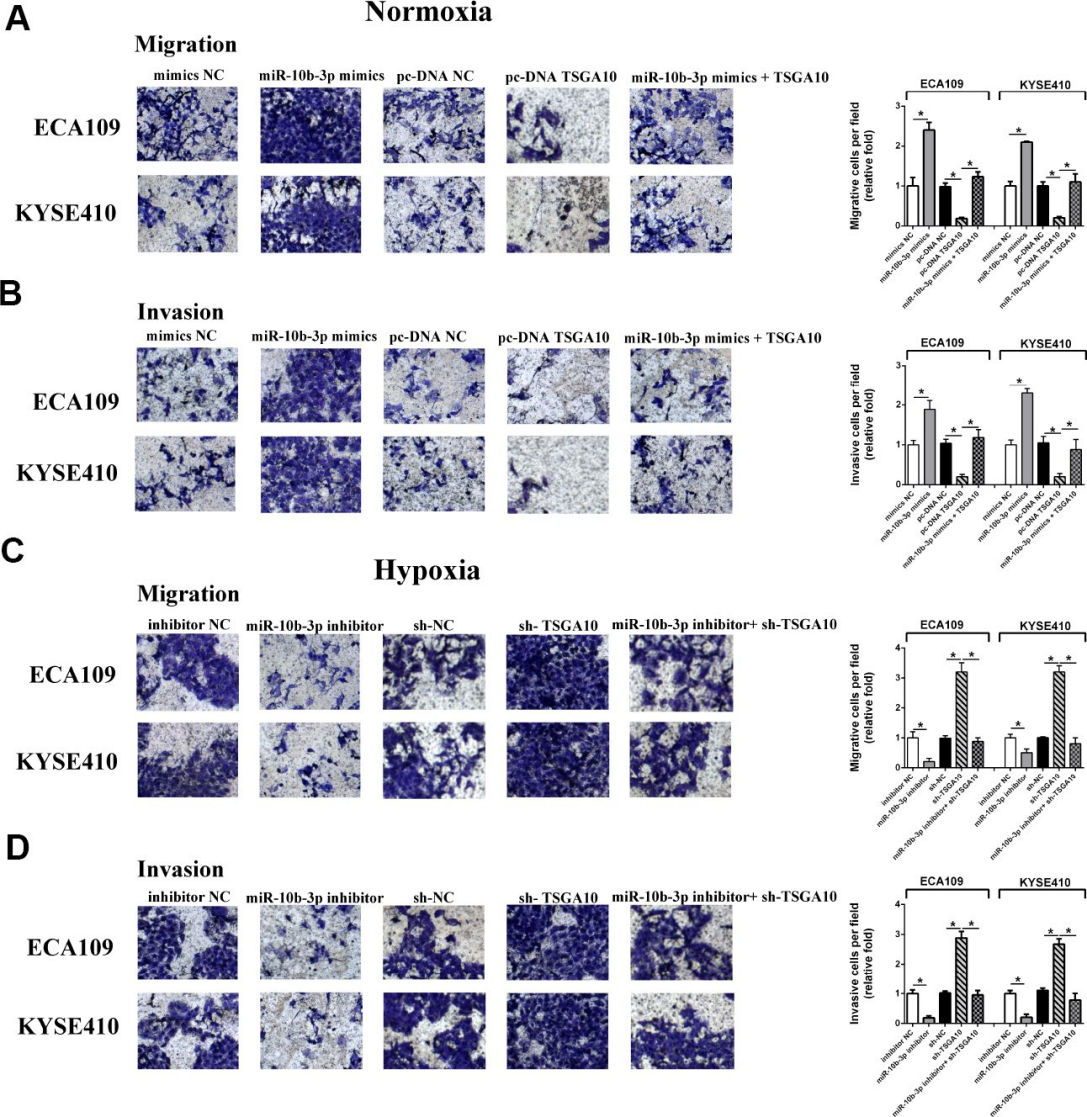
**Hypoxia-induced miR-10b-3p enhances ESCC cell migration and invasion.** (**A** and **B**) ECA109 and KYSE410 cells transfected with miR-10b-3p mimics or pc-DNA TSGA10 were seeded in serum-medium into Matrigel-free (**A**) or Matrigel-coated (**B**) upper chambers, while the lower chamber contained complete medium. After incubation for 20 hours under normoxic conditions, invading cells were fixed, photographed, and counted. (**C** and **D**) For hypoxia assays, ECA109 and KYSE410 cells transfected with miR-10b-3p inhibitor or sh-TSGA10 were seeded in serum-free medium into Matrigel-free (**C**) or Matrigel-coated (**D**) upper chambers, while the lower contained complete medium. After incubation for 20 hours under hypoxic condition, invading cells were fixed, photographed, and counted. *P < 0.05.

We next evaluated the influence of miR-10b-3p on ESCC cell migration and invasion. Using transwell assays, we observed that ectopic miR-10b-3p significantly enhanced the invasiveness of ESCC cells under normoxic conditions. However, up-regulation of TSGA10 diminished the effect of miR-10b-3p on ESCC cells (Figure 3A–3B). The invasiveness of ESCC cells was also enhanced by hypoxia; however, suppression of miR-10b-3p neutralized the effects of hypoxia. Rescue experiments showed that under hypoxic conditions TSGA10 knockdown restored the migration and invasion of ESCC cells that were inhibited by miR-10b-3p inhibition (Figure 3C–3D).

### Increasing miR-10b-3p enhances in-vivo tumor growth and distant metastasis

**Figure 4:**
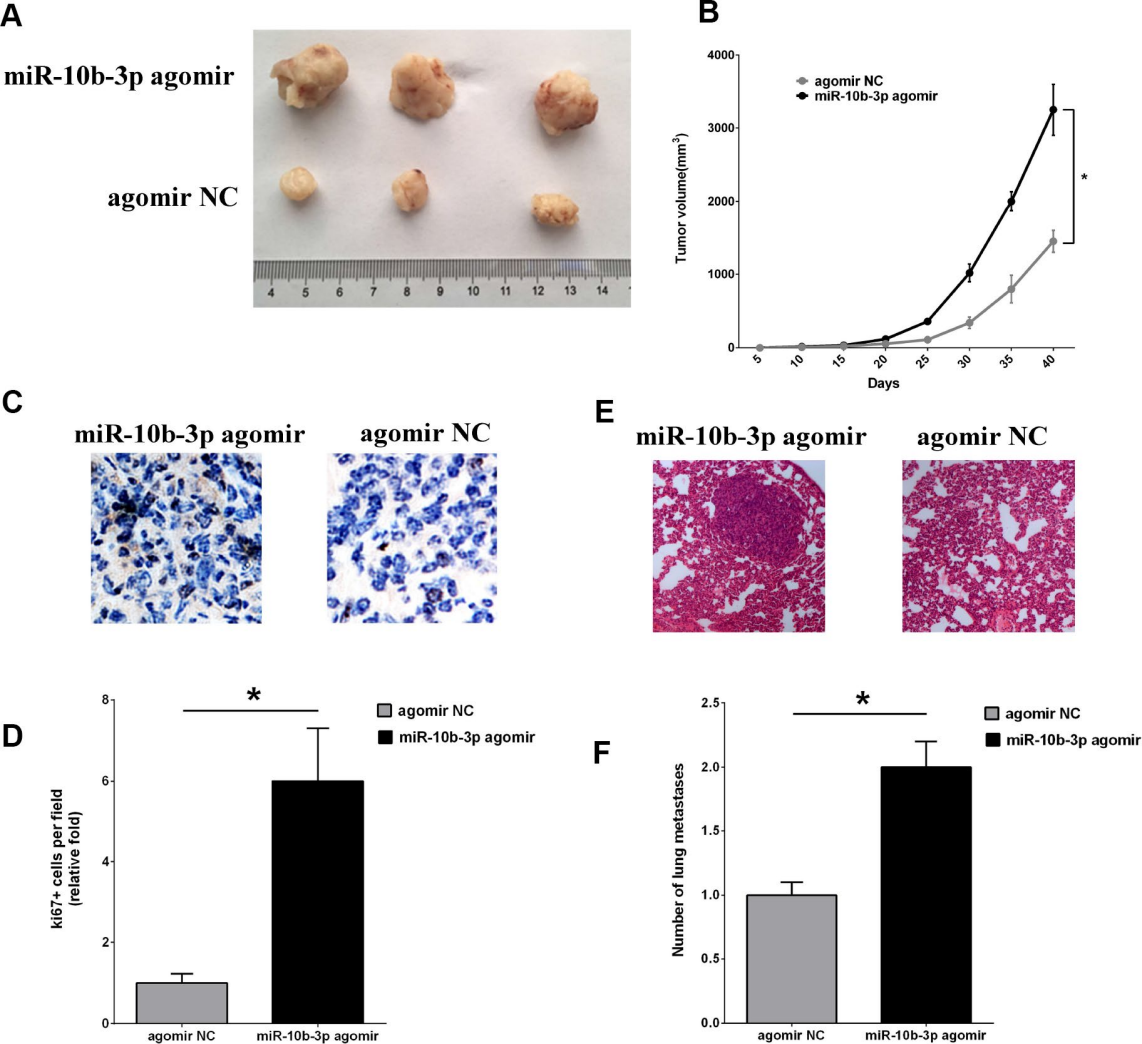
**MiR-10b-3p contributes to ESCC tumor growth and metastasis in vivo.** (**A**) An ESCC xenograft model in BALB/c nude mice was constructed using ECA109 cells. MiR-10b-3p agomir or agomir NC was injected into the tumor mass as indicated. After 40 days, tumors that developed were excised and photographed. (**B**) Tumor volumes were measured every 5 days and plotted. (**C** and **D**) To assess cell proliferation, tumor sections were immunostained for Ki-67 and photographed (**C**), after which Ki-67-positive cells were quantified (**D**). (**E**) Representative images of hematoxylin- and eosin-stained metastases in lungs. (**F**) Quantification of metastatic lung nodules. *P < 0.05.

To study the effects of miR-10b-3p in vivo, ECA109 cells were injected subcutaneously into the hind leg of BALB/c nude mice. Subsequent administration of miR-10b-3p agomir to the tumor significantly enhanced tumor growth as compared to that in the negative control group (agomir NC) (Figure 4A and 4B). Further analysis showed that the tumor volumes in the miR-10b-3p agomir group were significantly larger than those in the agomir NC group.

Finally, tumor cell proliferation was assessed based on immunohistochemical staining for Ki-67. The intensity of Ki-67 staining was significantly greater in tumors from the miR-10b-3p agomir group than the agomir NC group (Figure 4C and 4D). In addition, staining with hematoxylin and eosin revealed that there were more metastatic nodules present in the lungs of mice treated with miR-10b-3p agomir than agomir NC (Figure 4E and 4F).

## DISCUSSION

Evidence now suggests that miRNAs likely play key roles in the progression of ESCC [14]. For example, miR-145 and miR-133b, reportedly interact with fascin homolog 1 or actin-bundling protein, which leads to enhancement of cell growth and invasion in ESCC [15, 16]. On the other hand, miR-377 appears to restrain tumor growth and metastasis by targeting CD133 and VEGF in ESCC [17].

In solid tumors, hypoxia is a strong stimulus accelerating tumor glycolysis, angiogenesis, cell proliferation and metastasis through activation of HIFs [18, 19]. Among these mediators, it is well established that HIF-1α is expressed at only low levels under normoxia, but its levels increase dramatically upon induction of hypoxia [20]. Activated HIF-1α interacts with target genes by binding to their hypoxia response element (HRE) [21]. HIF-1α may exert its stimulatory. effects on cancer cells and tumor growth through activation of such target genes as VEGF, MMP-2 and BCL2 [22]. Studies of the interactions between HIF-1α and miRNAs have also shown that during prolonged hypoxia, HIF-1α regulates expression of miR-210, miR-155a, and miR-224 through binding to the HRE in the corresponding microRNA promoters [23–26]. Our study confirms that hypoxia leads to increased miR-10b-3p expression in ESCC cells and that this effect is mediated by HIF-1α.

We also explored the modulatory role of miR-10b-3p in ESCC cells under hypoxia. Our results show that suppressing miR-10b-3p attenuates the effects of hypoxia on ESCC cells. Moreover, we demonstrate that TSGA10 gene is a direct target of miR-10b-3p. TSGA10 is initially characterized as a testis-specific protein and tumor-associated antigen that facilitates angiogenesis and metastasis in various cancers [27]. The prognostic value of TSGA10 is also identified in acute myeloid leukemia and transitional cell carcinoma of the bladder [28, 29]. In ESCC, TSGA10 down-regulation is shown to be associated with a progressive clinical stage, and in vivo assays suggest TSGA10 knockdown significantly accelerates tumor growth and leads to larger tumor volumes [30]. Bao et al. studied the interaction between TSGA10 and miR-23a during angiogenesis and reported that in nasopharyngeal carcinoma exosome-derived miR-23a accelerates angiogenesis by targeting TSGA10 [27]. Our present results confirms that hypoxia-induced HIF-1α activation leads to upregulation of miR-10b-3p, which in turn facilitates tumor growth and metastasis by targeting TSGA10.

TSGA10 can bind to the HIF-1α C-terminal, resulting in HIF-1α inactivation [31]. Moreover, the interaction between TSGA10 and HIF-1α can prevent the transcriptional activity of HIF-1α and thus interfere with tumor growth, and angiogenesis [31]. The negative relationship between expression of TSGA10 and HIF-α subunits was also verified in both cervical and breast cancer [32]. In acute myeloid leukemia, the interaction between TSGA10 and HIF1-α leads to a decrease in VEGF secretion [29]. This suggests there may be a feedback loop among HIF1-α, miR-10b-3p and TSGA10 in ESCC cells, which deserves further exploration.

**Figure 5:**
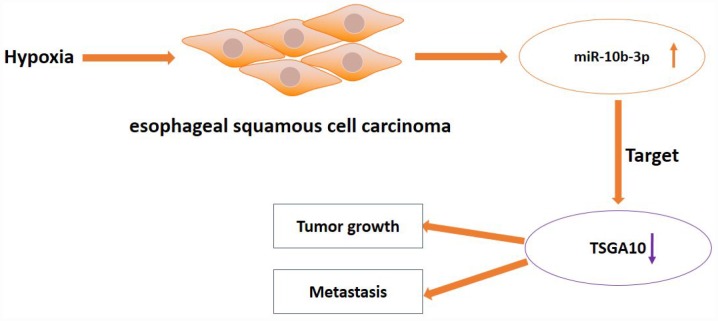
**Schematic diagram of hypoxia-induced miR-10b-3p promoting tumor growth and metastasis by targeting TSGA10 in ESCC.**

Taken together, our results demonstrate that hypoxia can increase expression of miR-10b-3p, which in turn targets TSGA10, leading to the promotion of ESCC tumor growth and metastasis (Figure 5).

## MATERIALS AND METHODS

### Cell lines and cell culture

ECA109 and KYSE410 cells were cultured in RPMI 1640 medium (Invitrogen, USA) supplemented with 10% fetal bovine serum (FBS) and 1% penicillin/streptomycin under a humidified atmosphere at 37°C. Normoxic conditions were created using a CO_2_ incubator with 5% CO_2_. For hypoxic conditions, the cells were maintained with 1% O_2_ and 5% CO_2_.

### RNA isolation and quantitative real-time polymerase chain reaction (qRT-PCR)

Trizol Reagent (Qiagen, USA) was applied to extract RNAs from ESCC cells. Isolated total RNA was used as a template for synthesis of single-stranded cDNA. A TaqMan MicroRNA Assay kit for hsa-miR-10b-3p (Thermofisher, USA) was used to quantify expression of miR-10b-3p. The qRT-PCR reactions were done using a Step One Plus real-time PCR system (Applied Biosystems). All of the reactions were run in triplicate.

### Overexpression and knockdown of miR-10b-3p

MiRNA mimics, mimics NC (negative control), miRNA inhibitors, and inhibitors NC were constructed by RiboBio (China) and then transfected into cells using Lipofectamine 2000 (Invitrogen, USA). Transfection efficiency was evaluated using qRT-PCR (Supplementary Figure 1).

### Plasmid construction

Plasmid including pcDNA-TSGA10, TSGA10 sh-RNA (sh-TSGA10), pcDNA-HIF-1α, HIF-1α sh-RNA (sh-HIF-1α), and their corresponding empty vector were constructed and purchased from RiboBio (China). Plasmids were transfected into cells using Lipofectamine 2000 Reagent (Invitrogen, USA).

### Western blot

Proteins were extracted using RIPA buffer, and protein concentrations were measured using a bicinchoninic acid (BCA) protein assay kit (Beyotime Biotechnology, China). Protein samples were then separated using the sodium dodecyl sulfate-polyacrylamide gel electrophoresis and transferred to polyvinylidene fluoride (PVDF) membranes. After blocking the membrane, it was incubated with the primary antibodies and corresponding secondary antibodies. Finally, the blots on the membranes were developed using Super ECL Plus (Beyotime, China) and quantified using image J software.

### CCK8 assays

For cell proliferation analysis, 5×10^3^ ESCC cells were seeded into 96-well plates and cultured under normoxic or hypoxic condition for 48 hours. Cell proliferation was then measured using a Cell Counting Kit-8 (CCK8) (Beyotime, China). Briefly, 20 μl of CCK8 solution were added to each well and incubated for 2 hours. The absorbance at 450 nm was then measured using a Microplate Reader (Bio-Rad, USA). Each assay was repeated at least three times.

### Colony formation assays

Transfected cells were plated into the 6-well plates (500–800 cells/well) and cultured under normoxic or hypoxic conditions for 10 days, replenishing the medium every 3 days. The colonies formed were then fixed using ethanol and stained with crystal violet solution.

### Migration and invasion assay

In transwell assays, 2.5 × 10^4^ ESCC cells were seeded in serum-free medium into upper transwell chambers (8 μm pore size; Costar) pre-coated with Matrigel (invasion assays) or without it (migration assays). The medium in lower chamber contained 10% FBS. After incubation for 20 hours, non-invading cells on the upside of the filters were scraped. Invading or migrating cells on the underside were fixed with ethanol, stained with crystal violet, and counted under an inverted microscope.

### Luciferase reporter assay

The 3′-UTR sequence (wild type (WT) or mutant (MUT)) of TSGA10 mRNA was amplified and cloned into the luciferase reporter vector. ECA109 and KYSE410 cells were then co-transfected with the 3′-UTR WT or 3′-UTR MUT vectors and miR-10b-3p mimics or mimics NC using Lipofectamine 2000. Luciferase activity was measured using a Dual-Luciferase Reporter Assay System (Promega, Fitchburg, WI, USA).

### In vivo animal study

The animal study was approved by the Animal Investigation Committee of The First Hospital of Qinhuangdao, and we complied with all relevant ethical regulations for animal testing and research.

To prepare a mouse xenograft model, 1×10^6^ ECA109 cells were injected subcutaneously into the right hip of 4- to 6-week-old BALB/c nude mice. MiR-10b-3p agomir (10 nmol) or agomir NC (10 nmol) (Ribobio, China) were injected into the tumor mass every 5 days. Tumor size was measured every 5 days, and the mice were sacrificed after 40 days.

### Immunohistochemistry

Paraffin-embedded sections of tumor tissues from nude mice were immunostained with anti-Ki-67 antibody (Servicbio, China) using the streptavidin peroxidase conjugate method. In addition, lung sections were stained with hematoxylin and eosin. Stained tumor and lung sections were visualized using an inverted microscope.

### Statistical analysis

Data were presented as the mean ± standard deviation (SD) or as the median with interquartile range. Graph Pad prism 7.0 was used for data analysis. Data were examined for normality using the Kolmogorov-Smirnov test. Differences between two groups were tested using Student’s t-test or the Mann-Whitney U test. Comparisons among more than two groups were made using one-way ANOVA followed by Holm-Sidak's multiple comparison tests or the Kruskal-Wallis test followed by Dunn's multiple comparison tests. Values of P<0.05 were considered significant. Each assay was repeated at least three times.

## Supplementary Material

Supplementary Figure 1
