# Supplementary material for: Hypoxia-induced microRNA-10b-3p promotes esophageal squamous cell carcinoma growth and metastasis by targeting TSGA10
Source: Aging (Albany NY). 2019 Nov 26;11(22):10374–84. doi: 10.18632/aging.102462 (PMC6914416; doi:10.18632/aging.102462)
Supplement: Supplementary Figure 1 [file aging-11-102462-s001..pdf]

SUPPLEMENTARY FIGURE

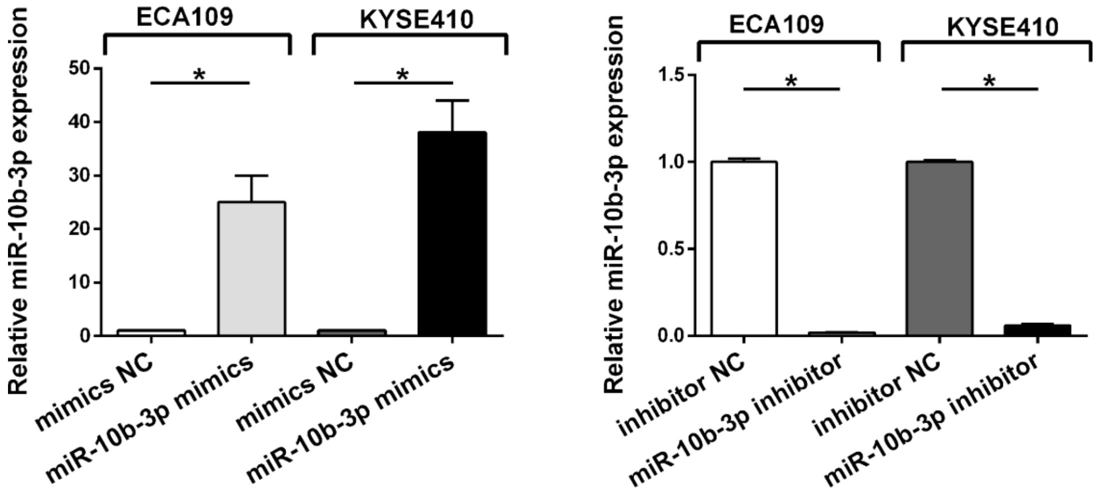

Supplementary Figure 1. Expression of miR-10b-3p in ESCC cells after transfection was measured using qRT-PCR.
